# Identity-by-descent mapping in a Scandinavian multiple sclerosis cohort

**DOI:** 10.1038/ejhg.2014.155

**Published:** 2014-08-27

**Authors:** Helga Westerlind, Kerstin Imrell, Ryan Ramanujam, Kjell-Morten Myhr, Elisabeth Gulowsen Celius, Hanne F Harbo, Annette Bang Oturai, Anders Hamsten, Lars Alfredsson, Tomas Olsson, Ingrid Kockum, Timo Koski, Jan Hillert

**Affiliations:** 1 Department of Clinical Neuroscience, Karolinska Institutet, Stockholm, Sweden; 2 Department of Mathematics, Royal Institute of Technology, Stockholm, Sweden; 3 KG Jebsen Centre for MS Research, Department of Clinical Medicine, University of Bergen, Bergen, Norway; 4 Norwegian Multiple Sclerosis Registry and Biobank, Department of Neurology, Haukeland University Hospital, Bergen, Norway; 5 Department of Neurology, Oslo University Hospital, Oslo, Norway; 6 Institute of Clinical Medicine, University of Oslo, Oslo, Norway; 7 Danish Multiple Sclerosis Center, Department of Neurology, Copenhagen University Hospital, Rigshospitalet, Copenhagen, Denmark; 8 Department of Medicine, Karolinska Institutet, Stockholm, Sweden; 9 Insitute for Environmental Medicine, Karolinska Institutet, Stockholm, Sweden

## Abstract

In an attempt to map chromosomal regions carrying rare gene variants contributing to the risk of multiple sclerosis (MS), we identified segments shared identical-by-descent (IBD) using the software BEAGLE 4.0's refined IBD analysis. IBD mapping aims at identifying segments inherited from a common ancestor and shared more frequently in case–case pairs. A total of 2106 MS patients of Nordic origin and 624 matched controls were genotyped on Illumina Human Quad 660 chip and an additional 1352 ethnically matched controls typed on Illumina HumanHap 550 and Illumina 1M were added. The quality control left a total of 441 731 markers for the analysis. After identification of segments shared by descent and significance testing, a filter function for markers with low IBD sharing was applied. Four regions on chromosomes 5, 9, 14 and 19 were found to be significantly associated with the risk for MS. However, all markers but for one were located telomerically, including the very distal markers. For methodological reasons, such segments have a low sharing of IBD signals and are prone to be false positives. One marker on chromosome 19 reached genome-wide significance and was not one of the distal markers. This marker was located within the *GNA11* gene, which contains no previous association with MS. We conclude that IBD mapping is not sufficiently powered to identify MS risk loci even in ethnically relatively homogenous populations, or that alternatively rare variants are not adequately present.

## Introduction

Multiple sclerosis (MS) is a chronic, lifelong, demyelinating disease, affecting primarily young adults. Genes are known to have an important role in the susceptibility to MS, indicated by a high value for heritability, *h*^2^, of 0.64 (CI: 0.36–0.76) in a recent study.^1^ By large-scale genotyping and case–control analyses, over 100 risk genes have been identified, which are estimated to explain less than one-third of the heritability.^2^ The identified risk genes so far are, owing to study design, common variants, and one possible explanation for the missing heritability could be that rare variants have an important role.

There are several methods for identification of rare gene variants that are important in the pathogenesis. The traditional approach to capture segments shared identical-by-descent (IBD) using affected families and linkage has generally been less productive in MS and in most complex genetic disorders. In MS, this approach is also limited by the low level of familial aggregation,^1^ making it difficult to obtain sufficient numbers of families with more than a few affected individuals. A possible exception to this failure may be the analysis of isolated or semi-isolated populations, such as Bothnian multicase MS families in Finland^3^ and an isolated Dutch population^4^ which both indicated the importance of specific genes, which however so far are not supported by data from the large international case–control studies.

Population-based linkage analysis (PBLA) is an approach in which data from SNP genotyping are used to detect segments with IBD shared more often among cases than among controls,^5^ a method hypothesized to catch signals of rare variants. Here, we apply a PBLA approach on a data set of Scandinavian MS patients that was included in a previously published genome-wide association screen.^6^ Although Scandinavia is not what is traditionally seen as an isolate, it is a population that clusters very closely in principal component analysis.^6^ It can therefore be regarded as a genetically relatively homogenous population and because of the high prevalence of MS, it might be well suited for PBLA.

A number of different methods applicable to PBLA have been developed. One of the first softwares was PLINK's segmental sharing algorithm published in 2006,^5^ which used a Hidden Markov Model (HMM)^7^ to detect segments shared by descent. More recent approaches include GERMLINE,^8^ which uses a dictionary approach, and BEAGLE IBD,^9^ also using an HMM methodology. Beagle IBD was shown to be more accurate but in turn quite slow in running time. Browning and Browning^10^ developed a new method, fastIBD, which outperformed PLINK substantially in accuracy and power, and was able to detect segments among more distantly related individuals. In a recent study by Gauvin *et al*,^11^ fastIBD was found to be more reliable than GERMLINE, PLINK and two other methods^12, 13^ when compared using real data. Recently, Browning and Browning^14^ published a further update of their method, refined IBD, in which they abandoned the HMM's and used a dictionary approach, like GERMLINE, but combined it with a probabilistic assessment of the segments being shared IBD *versus* not being shared IBD, and thus gaining even more power. For segments of shorter length, refined IBD detected a higher number of segments than fastIBD,^14^ and may therefore be even more appropriate for outbred populations such as those in this project.

Common for all the above-mentioned methods is that, they all use sharing identical-by-state (IBS) to estimate sharing IBD. As frequencies of loci can be significantly different among populations, stratification of population structure within the data is crucial to avoid spurious associations. Attention to detection and removal of outliers and/or close relatedness is therefore an important step to entail a data set suitable for analysis. In order to estimate IBD from IBS, these steps must be given careful attention.

## Materials and methods

### Genotyping and quality control

In all, 2106 MS patients of Nordic origin from Sweden (*n*=713), Norway (*n*=1030) and Denmark (*n*=363), and 624 controls matched on the Swedish sample were genotyped on the Illumina Human Quad 660 chip (Illumina, San Diego, CA, USA) and quality controlled as described elsewhere^6^ (data accessible at https://www.ebi.ac.uk/ega/studies/EGAS00000000101). From this quality control, 91 individuals were excluded due to genotyping error and/or close relationship.

An additional 678 controls for breast cancer patients treated in the Stockholm area,^15^ typed on Illumina 1M (Illumina), and 674 controls for Swedish patients with myocardial infarction,^16, 17^ typed on Illumina HumanHap 550k (Illumina), were also added. The data are available upon request from http://www.karmastudy.org (breast cancer) and http://procardis.org (myocardial infarction), respectively.

An additional quality control, after re-calling the genotypes for the additional controls and combining the two data sets, was performed with PLINK using a minor allele frequency of 0.05, Hardy–Weinberg equilibrium of 1e-6, a missingness per individual of 0.07, as required by Beagle,^14^ and a missingness per marker of 0.1, left 441 731 markers in the analysis.

### Outlier analysis

The smartPCA algorithm of the EIGENSTRAT package^18^ was used for calculating the principal component (PC) vector and removing outliers. The outlier-removal process involved two stages, utilizing as input data the first six PCs. In the first stage, sample pairwise Euclidean distances were used to calculate the average distance of a sample to each of its 10 nearest neighbors. This information gives the density of local clustering along PCs, and an arbitrary cutoff (distance of 0.15) was used to determine the main cluster(s), thereby taking into account the sparseness of the sample distribution. In the second stage, samples in the included cluster(s) were required to also have 9 of 10 the nearest neighbors inside the cluster. This ensures that only samples at the interior of clusters are included, and less tightly included samples approaching the cluster boundary are omitted to ensure a more homogeneous composition. A final set of 3953 individuals remained after the exclusion of outliers. Scripts for R^19^ and MATLAB^20^ for the outlier analysis can be found on http://kirc.se.

### Transformation of data sets

The data set was transformed from PLINK's .ped and .bed format to Beagle's .bgl format using linkage2beagle.jar, and later from .bgl to .vcf using beagle2vcf.jar from the Beagle utility programs.

### Identification of IBD segments

A pre-release version of BEAGLE 4.0 was used for detection of the segments. The ibdtrim parameter was set to 25. The centiMorgan distances for the map-file were interpolated using the Beagle utility program base2genetic.jar and build 36 of the human genome project.

### IBD mapping

A Java program was written to convert the format from refined IBD to fastIBD (available on http://kirc.se) and the scripts for IBD mapping published by Sharon Browning^21^ were used to perform the IBD mapping. The threshold for genome-wide significance was estimated through the permutation analysis provided by Sharon Browning's script using 5 million permutations. When calculating the *P*-values in the permutation analysis, it was corrected for the average genome-wide sharing.

The analysis was run on a two-server computational cluster, where each machine was equipped with two Intel Xeon E5-2660 2.20 GHz processors, 128 GB RAM with Scientific Linux 6.3 as operating system and SLURM as resource management system.

Segments with a LOD score of <3 and a length shorter than 1 cM were excluded before calculating the genome-wide average and performing the permutation analysis.

The threshold for genome-wide significance was set as the 0.05 percentile of the distribution for the permutation *P*-values.

## Results

**Figure 1:**
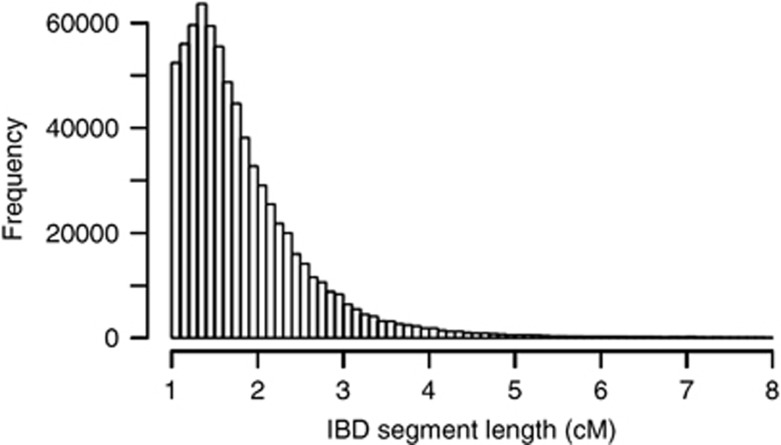
Plot of the distribution of the lengths of the segments.

A histogram over the frequency of lengths of detected chromosomal segments can be seen in Figure 1. The distribution approximated a Pareto distribution with the mean lengths of segments slightly above 1 cM.

**Figure 2:**
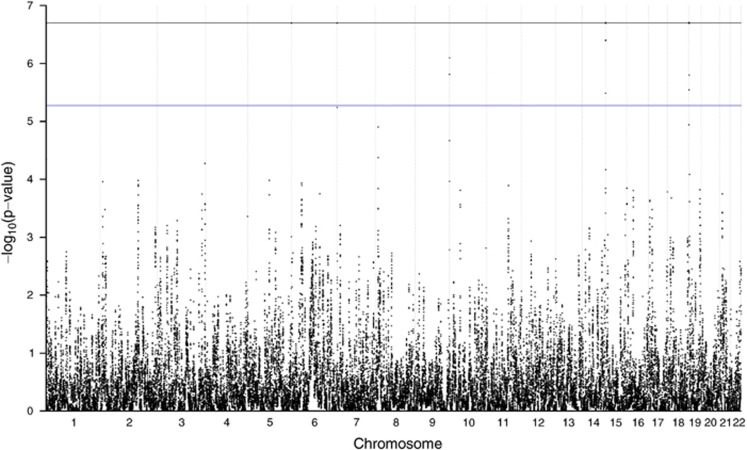
Permutation analysis before filtering. Blue line indicates genome-wide threshold and black line is the minimal permutation *P*-value. The full colour version of this figure is available at *European Journal of Human Genetics* online.

**Figure 3:**
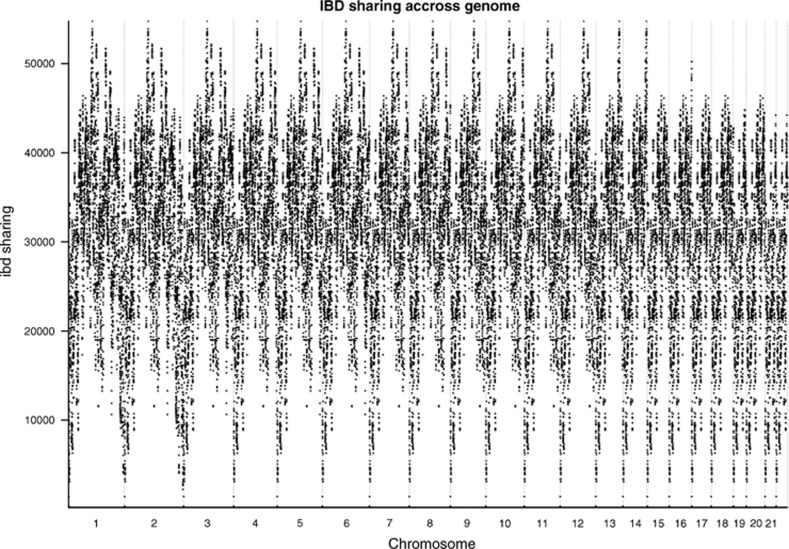
Pattern for IBD sharing per chromosome.

The permutation analysis showed significant peaks of the segment shared IBD at the ends of chromosomes 5, 9 and 14 and in the beginning of chromosomes 1, 7, 15 and 19 (Figure 2). Determining the correct end points of segments is a known difficulty while identifying segments,^14^ and IBD detection at the end of chromosomes is lower. This can be seen in Figure 3 where the IBD sharing between all pairs of individuals is shown. This may inflate significance estimations for segments identified to be shared more often between affected individuals. Thus, the fact that most associated segments were located telomerically, strongly suggests that their identification was an artifact of the method. Therefore, a filter removing signals in regions with the lowest 10% of IBD detection was added. To this end, we calculated how many segments spanned each accepted marker and estimated the 10th percentile from the distribution.

**Figure 4:**
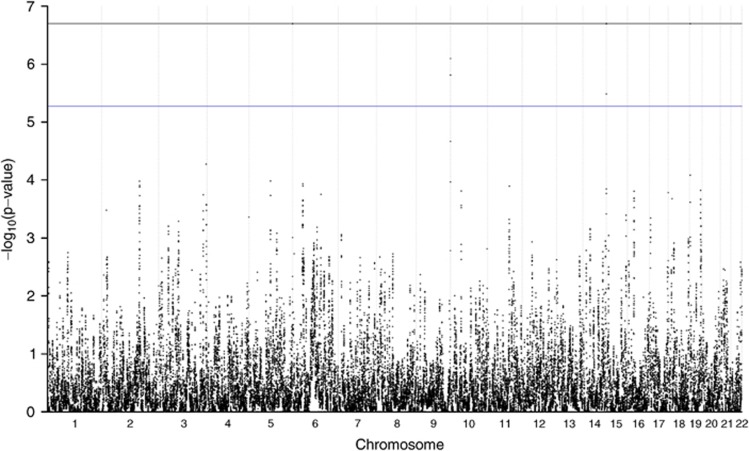
Permutation analysis after filtering out regions with low IBD sharing. Blue line indicates genome-wide threshold and black line is the minimal permutation *P*-value. The full colour version of this figure is available at *European Journal of Human Genetics* online.

After applying the filter, the peaks on chromosomes 5, 9, 14 and 19 remained (Figure 4). The signal on chromosome 19 was the only genome-wide significant hit that was not in a telomeric position. Here, a single marker (RS8092) reached significance in the permutation analysis, whereas the flanking markers did not (RS4806907 and RS1682809). The significant marker was located in the last exome of transcript 001 of gene *GNA11*.

## Discussion

We aimed at reusing data from a published GWAS for assessing chromosomal segments shared IBD between patients and controls to achieve a PBLA of a genetically relatively homogenous cohort of individuals from Scandinavia. When applying the statistical methodology of choice on over 2000 patients and as many controls, only one marker was found to reach genome-wide significance. None of the over 100 previously identified MS risk gene loci indicated an increased sharing of haplotypes between patients.

Assuming that lack of power contributed to the lack of significant findings in this study, we attempted to infer the power calculations described for refined IBD to our settings.^14^ This suggested that, theoretically, we may have reached a power >50% with our sample size of *n*=2000 using a LOD of 3 as a cutoff to identify segments of 1 cM length or greater. However, extensive simulations would be required to definitively assess the real power of the analysis.

Another explanation for the results could be that, despite removing outliers and retrieving a homogenous data set, the population might still be too outbred to find significant results using this type of analysis. In Sweden, there are a few identified high MS prevalence clusters such as Lysvik in the west^22^ and Överkalix in the north.^23^ Even if rare genetic variants explain these clusters and samples from these and other possible clusters are included, such variants may have been missed due to dilution when including them in the larger material.

A further speculation would be that rare variants are less important as genetic risk factors for MS in comparison with the common variants responsible for the previously identified genetic effects in MS.^2, 6^

Although major improvements have been made in accuracy and speed of haplotype sharing algorithms compared with previous methods, there are still slight problems with the refined IBD method. One of the problems is the difficulty to avoid false positives in regions with low coverage of IBD such as at ends of chromosomes, an artifact seen in this study. This prompted us to the filtering out of regions with low IBD, which left one marker that was not in a telomeric region. This variant maps to *GNA11*, which codes for a guanine nucleotide binding protein (G protein), alpha 11 (Gq class), a gene not previously associated with MS and located on 19p13.3. The closest previously published MS-associated gene is *TNFSF14* that is located some 3 MBp centromeric. Already in 2005, a linkage peak for MS was reported in 19p13 that acted independently from the HLA locus;^24^ however, it is not specified as to where on 19p13 this effect was seen. In a paper in 2009, a microsatellite marker on 19p13 was associated to disease outcome,^25^ but upon our inspection in a later build of the genome reference, it appears that this marker is more likely to reside at 19q13, an area with several published associations from both linkage and association analysis.^26, 27, 28, 29^ Thus, there is weak prior evidence for an importance of this locus in MS.

Earlier in 2013, a paper performing PBLA in MS using fastIBD was published by a group in Australia,^30^ reporting a peak at the end of chromosome 19. A different method for post-processing of the segments was used and no filtering out of regions with general low IBD detection was made. This paper also presented a significant finding in the HLA region, the most strongly acting gene region in MS. The most straightforward explanation for the lack of signal in HLA in our study is that there are no rare variants in this region of the Swedish population. This would indicate that the HLA associations reported in the literature are due to common variations and not due to, for example, sequence variations within the associated alleles. Other possible explanations could be the shorter length of segments in the region, which in our analysis were filtered out already during the segment identification phase when they did not reach the 1 cM threshold.

There are a few potential problems to consider when running PBLA, and parameter settings can be difficult. The extensive amount of hardware and computational time for the analysis introduces difficulty and parameter sweeping is not an option. The extensive permutation analysis performed in this study gave a more accurate estimate for the genome-wide threshold. Nevertheless, it took months to run despite the use of a reasonably fast computational cluster with a suitably large amount of hardware. There are other ways of obtaining an estimate for this threshold,^10^ but they are less accurate and require time and computer power to perform the analysis.

## Conclusion

Detecting segments shared by descent is a very intriguing method; however, it is still very new and to date not well tested. Much has happened since the first version of PLINK's segmental sharing algorithm was published in 2006, and methods are now both faster and more accurate with higher power.^10^ Using available methods, the outcome of analysis was largely negative, with one significant marker shared more frequently in haplotypes estimated to be identical by descent among MS case–case pairs in a Scandinavian population.
